# Artificial Intelligence in Intensive Care Medicine: Bibliometric Analysis

**DOI:** 10.2196/42185

**Published:** 2022-11-30

**Authors:** Ri Tang, Shuyi Zhang, Chenling Ding, Mingli Zhu, Yuan Gao

**Affiliations:** 1 Department of Intensive Care Medicine Renji Hospital Affiliated to Shanghai Jiao Tong University School of Medicine Shanghai China

**Keywords:** intensive care medicine, artificial intelligence, bibliometric analysis, machine learning, sepsis

## Abstract

**Background:**

Interest in critical care–related artificial intelligence (AI) research is growing rapidly. However, the literature is still lacking in comprehensive bibliometric studies that measure and analyze scientific publications globally.

**Objective:**

The objective of this study was to assess the global research trends in AI in intensive care medicine based on publication outputs, citations, coauthorships between nations, and co-occurrences of author keywords.

**Methods:**

A total of 3619 documents published until March 2022 were retrieved from the Scopus database. After selecting the document type as articles, the titles and abstracts were checked for eligibility. In the final bibliometric study using VOSviewer, 1198 papers were included. The growth rate of publications, preferred journals, leading research countries, international collaborations, and top institutions were computed.

**Results:**

The number of publications increased steeply between 2018 and 2022, accounting for 72.53% (869/1198) of all the included papers. The United States and China contributed to approximately 55.17% (661/1198) of the total publications. Of the 15 most productive institutions, 9 were among the top 100 universities worldwide. Detecting clinical deterioration, monitoring, predicting disease progression, mortality, prognosis, and classifying disease phenotypes or subtypes were some of the research hot spots for AI in patients who are critically ill. Neural networks, decision support systems, machine learning, and deep learning were all commonly used AI technologies.

**Conclusions:**

This study highlights popular areas in AI research aimed at improving health care in intensive care units, offers a comprehensive look at the research trend in AI application in the intensive care unit, and provides an insight into potential collaboration and prospects for future research. The 30 articles that received the most citations were listed in detail. For AI-based clinical research to be sufficiently convincing for routine critical care practice, collaborative research efforts are needed to increase the maturity and robustness of AI-driven models.

## Introduction

### Background

Artificial intelligence (AI) refers to a system that mimics human intelligence as characterized by the ability to perceive, reason, discover meaning, generalize, draw lessons from past experience and solve problems, or make decisions [1]. Machine learning (ML), natural language processing, and capability to visualize and recognize objects (computer vision) are all commonly used AI technologies [2].

**Figure 1:**
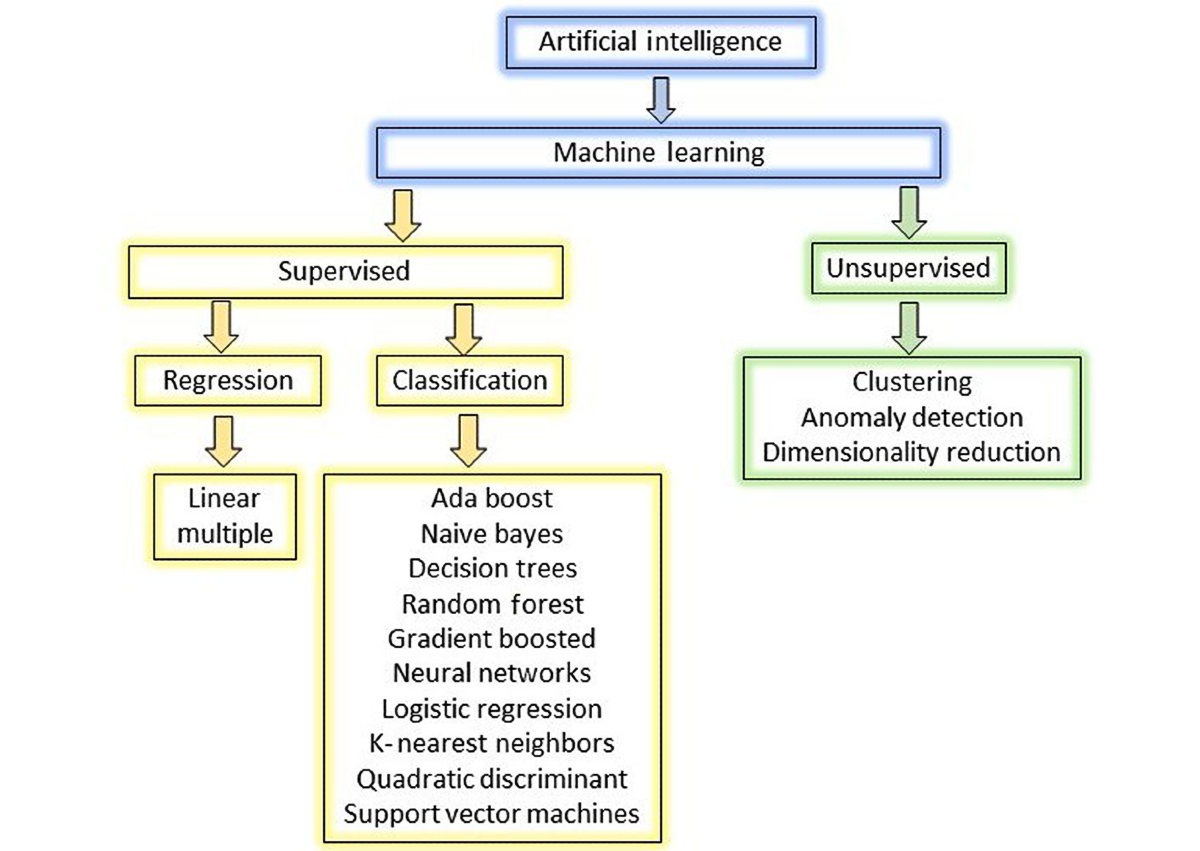
Machine learning is a branch of artificial intelligence encompassing two major approaches: supervised and unsupervised learning. Shown under each branch are algorithm types used in model development.

ML is the dominant technique for implementing Al systems. ML refers to the science of programming computers that use statistical analysis techniques to create algorithms to learn from data [1]. Because it uses statistical models and algorithms to evaluate enormous training data sets, it is also referred to as “programming with data.” Supervised and unsupervised frameworks are two different types of ML (Figure 1).

AI has been used in the medical field in molecular biology, bioinformatics, and medical imaging and to support population health management, provide tailored diagnosis and treatment, monitor patients, guide surgical care, and predict health trajectories [1,3,4].

The intensive care unit (ICU) is the most suitable ward among all the hospital wards to begin the transition to big data and the application of AI in research and even clinical practice in the near future. In ICUs, patients are closely monitored to detect physiological changes associated with deterioration that might require an appropriate reevaluation of the treatment plan. Nursing staff closely monitor patients in the ICU by charting neurological status, input, and output (including medication administration), etc. Bedside monitors facilitate this and continuously stream large amounts of data [5]. With advances in computer science, it has become possible to integrate and archive data in clinical documentation from various information systems and build a comprehensive system that is later transformed into a research database. Large public ICU databases include eICU and MIMIC databases. Open access to these databases encourages the use of AI technology in clinical research in intensive care medicine and the development of decision support tools.

The application of AI in critical care mainly involves disease diagnosis, prediction of disease progression (clinical deterioration), and characterization of specific disease phenotypes or endotypes in sepsis, septic shock, and acute respiratory distress syndrome (ARDS), etc.

Bibliometric study is a quantifiable informatics technique that analyzes the academic literature [6,7]. A general, quantitative, and qualitative overview of a certain topic can be provided via bibliometric analysis. It specifically identifies the most active authors, organizations, publications, influential studies, and international collaborations [7].

### Goal of This Study

Although there has been a growing interest in critical care–related AI research, the literature is still lacking in comprehensive bibliometric studies that measure and analyze scientific publications globally. In this study, we aimed to (1) provide a holistic view of the research trends in AI application in ICUs; (2) highlight trending research topics in AI-related research focused on health care in ICUs; (3) highlight the contributions of prolific authors, leading countries, and the most productive academic institutions; and (4) provide an insight into potential collaboration and research directions in the future [8].

## Methods

A bibliometric analysis study uses a mechanistic method to comprehend the global research trends in a certain field based on the outputs of the academic literature database. This approach distinguishes bibliometric analysis from reviews that are primarily designed to discuss the most recent advancements, challenges, and future directions of a particular topic [8].

### Ethical Considerations

Ethics committee permission was not required, as this study was a retrospective bibliometric analysis of the existing published studies.

### Data Source and Search Strategy

Data mining was conducted on March 18, 2022, using the Scopus database. Scopus is recognized as the largest abstract and citation database of peer-reviewed literature covering a wide range of subjects [8]. This study conducted a search for articles mentioning artificial intelligence or AI-related terms (neural network*, machine learning, deep learning, or natural language processing) and intensive care or ICU-related terms (critical care, critically ill, high dependency, or ICU) in the title, abstract, and keywords. The oldest publication dates to 1986, and the more recent ones are from 2022. The reproducible query string used for the search was: TITLE-ABS-KEY (“artificial intelligence” OR “neural network*” OR “machine learning” OR “deep learning” OR “natural language processing”) AND (“intensive care” OR “critical care” OR “critically ill” OR “high dependency” OR “ICU”) SEARCHED ON 18, March 2022.

### Screening Strategy

The query string yielded 3619 documents. From those, only articles (2050/3619, 56.64%) were included (Table 1). A total of 4 duplicate articles were removed by using Stata (version 17; StataCorp) for data cleaning. The papers analyzed were restricted to those that (1) focused on intensive care medicine and (2) involved AI technologies. Two coauthors (SZ and RT) reviewed the titles of all studies as a pilot screening and removed irrelevant articles. Papers from the preliminary searches were categorized into include, exclude, or unsure. The abstracts and keywords of papers marked as unsure were further screened by 3 authors (SZ, RT, and MZ) and discussed until a consensus was reached in team meetings.

After screening the titles of all articles and abstracts, when necessary, 848 articles were excluded either because they did not focus on intensive care medicine or because they did not involve AI technologies. Finally, 1198 papers were included in the bibliometric analysis.

**Table 1:** The distribution of the bibliographic records by document type (N=3619).

Type of document	Frequency, n (%)
Article	2050 (56.64)
Conference paper	972 (26.85)
Review	241 (6.65)
Editorial	126 (3.48)
Note	66 (1.82)
Conference review	59 (1.6)
Letter	57 (1.57)
Book chapter	31 (0.85)
Short survey	9 (0.21)
Data paper	4 (0.1)
Erratum	4 (0.1)

### Statistical Analysis

#### Overview

We used VOSviewer (version 1.6.18; Centre for Science and Technology Studies, Leiden University), a software tool for constructing and visualizing bibliometric maps, for bibliometric network visualization. The citation, bibliographical, and author keyword information of 1198 articles were exported to VOSviewer. The countries, authors, institutions, or keywords were included as objects of interest when creating maps using VOSviewer. We computed the growth rate of publications, research keywords, and publication patterns (countries, institutions, and journals). Bibliometric analyses were performed according to the instructions provided in the VOSviewer user manual [9].

#### Publication Output and Growth of Research Interest

The publication years were sorted, and the number of publications each year was counted using Stata 17.

The growth rate of publications over time was computed using the following compound annual growth rate formula:

Growth rate = ([number of publications in the last year or number of publications in the first year]1/(last year − first year) − 1) × 100 [10-12].

#### Preferred Journals

We used the citation analysis function of VOSviewer and set the unit of analysis as “sources.” Of the 443 sources (journals), 44 (9.9%) had >5 publications in total on AI in intensive care medicine. Journals were sorted according to the number of publications. We listed the number of citations, an important index of the degree of attention and influence of the published papers [13,14]. CiteScore 2020 was obtained from the Scopus Preview website [15].

#### Leading Countries, International Collaboration, and Top Institutions

The citation trends of the top 10 most productive countries, top 15 most productive journals, and top 15 most productive research institutions were analyzed. The frequency and percentage of publications or citations in each country, journal, and institution were computed. This information was provided by Scopus and analyzed using the citation and coauthorship functions in VOSviewer. In the coauthorship analysis, the country-to-country link strength showed the number of publications coauthored by 2 linked countries. We created a thesaurus file to merge same institutions with different name variants.

Google Mymaps [16] was used for world map drawing. Using information from the International Monetary Fund’s World Economic Outlook, the gross domestic product of the countries was estimated to ascertain whether the economic power of the countries had an impact on the productivity of publications [17].

#### Author Keywords

A total of 2267 keywords from 892 (74.5%) articles were analyzed for author co-occurrence. Owing to the lack of author keyword information, the remaining 306 (25.5%) articles were excluded. A thesaurus file was created to merge synonymic single words and congeneric phrases. For example, *coronavirus*, *coronavirus disease 2019*, and *sars-cov-2* were merged into 1 keyword and relabeled as *covid-19*. We identified high-frequency keywords and classified them into 3 categories: diseases, technology, and function.

## Results

### Publication Output and Growth of Research Interest

**Figure 2:**
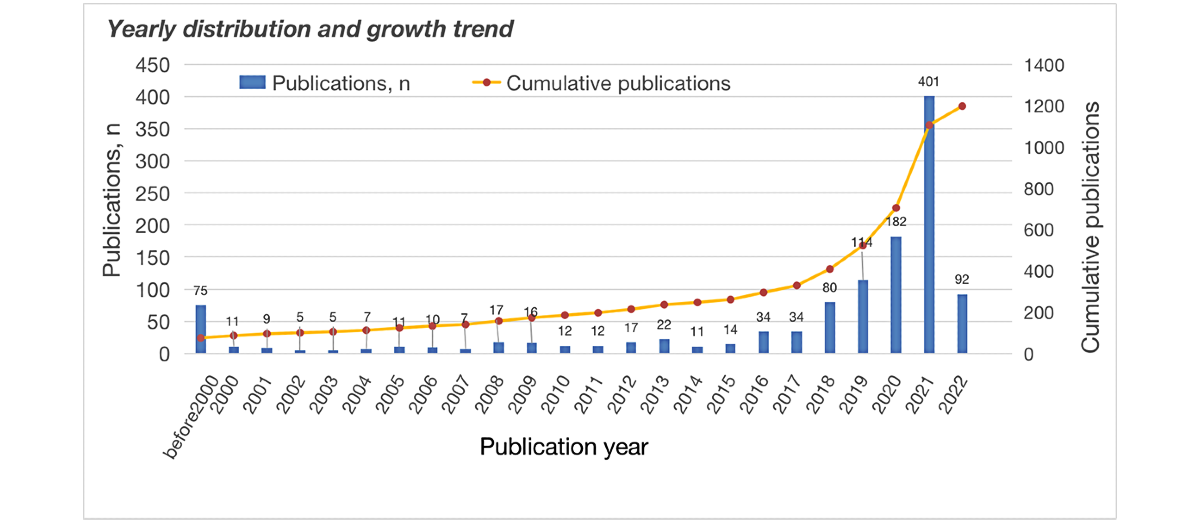
The annual and cumulative numbers of research articles on artificial intelligence in intensive care in Scopus from 1986 to 2022.

In all, 1198 research articles were published in 36 years (1986-2022; Figure 2). The oldest publication dates to 1986 [18].

It is suggested that a strong interest in AI in intensive care medicine started from 2018 when the annual growth rate increased by 135.3%. Since then, there has been a steep increase in annual publications, which has caused the cumulative number of publications to increase rapidly. The growth rate was 3.93% from 2011 to 2015 52.1% from 2016 to 2020, and the growth rate doubled last year to 120.3%. The number of publications increased steeply between 2018 and 2022, accounting for 72.53% (869/1198) of all the included papers.

Most articles (61.1%, 732/1198) were open-access articles that could be accessed for free. An article is likely to receive more citations if it is published in an open-access journal.

### Preferred Journals

Our results revealed that the top 15 most productive journals were owned by 9 different publishers (Table 2). The most productive journal was *Artificial Intelligence in Medicine* with 42 articles, covering 3.5% of the total publications, followed by *Critical Care Medicine* (40, 3.3%), *PLoS ONE* (35, 2.9%), and *Scientific Reports* (33, 2.8%). *Critical Care Medicine*, a Lippincott Williams and Wilkins journal, received the highest number of citations—1166. One of their articles, “An Interpretable Machine Learning Model for Accurate Prediction of Sepsis in the ICU,” published in 2018, was the most cited article, with 247 citations.

A total of 9 journals had a CiteScore of ≥5 according to the CiteScore 2020 report. *Critical Care Medicine* (CiteScore 12.7) and *JMIR Medical Informatics* (CiteScore 1.59) had the highest and lowest CiteScores, respectively. Although ranked 7th with 26 articles in Scopus, the total number of citations and number of citations per document of *Frontiers in Medicine* were significantly lower than those of other journals.

**Table 2:** The top 15 most productive journals on artificial intelligence in critical care research with their most cited article.

	TP^a^ (N=1198), n (%)	TC^b^, n	CiteScore 2020	Ranking based on citation	Citation per document	Most cited article	Times cited, n	Publisher
*Artificial Intelligence in Medicine*	42 (3.51)	983	8	2	23.40	A Working System for the Automated Control of Assisted Ventilation in ICUs	84	Elsevier
*Critical Care Medicine*	40 (3.34)	1166	12.7	1	29.15	An Interpretable Machine Learning Model for Accurate Prediction of Sepsis in the ICU	249	Lippincott Williams and Wilkins Ltd
*PLoS ONE*	35 (2.92)	412	5.3	7	11.77	Machine Learning Models for Early Sepsis Recognition in the Neonatal Intensive Care Unit Using Readily Available Electronic Health Record Data	48	Public Library of Science
*Scientific Reports*	33 (2.75)	315	7.1	8	9.55	Prediction of Ventricular Tachycardia One Hour Before Occurrence Using Artificial Neural Networks	56	Springer Nature
*Computer Methods and Programs in Biomedicine*	26 (2.17)	241	7.7	11	9.27	An Empirical Evaluation of Deep Learning for ICD-9 Code Assignment Using MIMIC-III Clinical Notes	36	Elsevier
*Critical Care*	26 (2.17)	470	10.1	4	18.08	Development and Validation of a Novel Molecular Biomarker Diagnostic Test for the Early Detection of Sepsis	96	BioMed Central Ltd
*Frontiers in Medicine*	26 (2.17)	57	4.1	15	2.19	A Machine Learning-Based Prediction of Hospital Mortality in Patients With Postoperative Sepsis	14	Frontiers Media SA
*BMC Medical Informatics and Decision Making*	25 (2.09)	233	3.9	12	9.32	A Comparative Analysis of Multi-Level Computer-Assisted Decision Making Systems for Traumatic Injuries	33	Springer Nature
*Computers in Biology and Medicine*	25 (2.09)	464	7.3	6	18.56	A Computational Approach to Early Sepsis Detection	116	Elsevier
*Journal of Clinical Monitoring and Computing*	25 (2.09)	300	3.7	10	12.00	Using Physiological Models and Decision Theory for Selecting Appropriate Ventilator Settings	72	Springer Nature
*IEEE Journal of Biomedical and Health Informatics*	21 (1.75)	116	10.2	13	5.52	Multi-Sensor Fusion Approach for Cuff-Less Blood Pressure Measurement	32	IEEE
*International Journal of Medical Informatics*	21 (1.75)	468	7.1	5	22.29	User-Centered Design Techniques for a Computerised Antibiotic Decision Support System in an Intensive Care Unit	61	Elsevier
*JMIR Medical Informatics*	19 (1.59)	312	2.9	9	16.42	Prediction of Sepsis in the Intensive Care Unit With Minimal Electronic Health Record Data: a Machine Learning Approach	203	JMIR Publications Inc
*Journal of Biomedical Informatics*	19 (1.59)	522	8.1	3	27.47	Reducing False Alarm Rates for Critical Arrhythmias Using the Arterial Blood Pressure Waveform	151	Academic Press Inc
*IEEE Access*	17 (1.42)	112	4.8	14	6.59	Predicting Complications in Critical Care Using Heterogeneous Clinical Data	25	IEEE

^a^TP: total publications.

^b^TC: total citations.

### Leading Countries, International Collaboration, and Top Institutions

**Figure 3:**
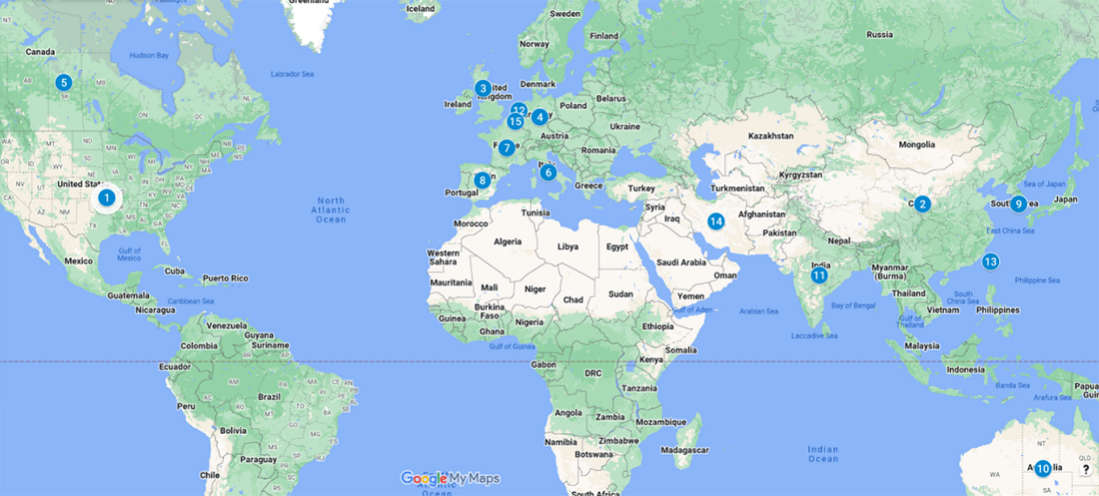
World map of the top 15 most productive countries based on publications on artificial intelligence in intensive care units.

The top 15 most productive countries contributing to the growth of AI in critical care research activities globally are listed in Table 3 and Figure 3. The United States was the leading country with 488 publications, accounting for 40.73% of all publications (1198) worldwide. With one-third of the total publications in the United States, China was the second most productive country (173/1198, 14.44%).

**Table 3:** The distribution of the bibliographic records by top 10 (by quantity) countries.

Country	Rank based on total output	Output (N=1198), n (%)	Citations (N=18,876),n (%)	Rank based on citations	Citation per document	GDP^a^ rank
United States	1	488 (40.7)	8678 (46)	1	17.78	1
China	2	173 (14.4)	1294 (6.9)	3	7.48	2
United Kingdom	3	116 (9.7)	2765 (14.7)	2	23.84	6
Germany	4	62 (5.2)	873 (4.6)	5	14.08	4
Canada	5	60 (5)	740 (3.9)	6	12.33	8
Italy	6	58 (4.8)	386 (2)	12	6.66	9
France	7	52 (4.3)	905 (4.8)	4	17.40	7
Spain	8	47 (3.9)	351 (1.9)	13	7.47	15
South Korea	9	46 (3.8)	442 (2.3)	10	9.61	12
Australia	10	41 (3.4)	520 (2.8)	8	12.68	13
India	11	39 (3.3)	495 (2.6)	9	12.69	5
Netherlands	12	39 (3.3)	534 (2.8)	7	13.69	19
Taiwan	13	35 (2.9)	319 (1.7)	14	9.11	21
Iran	14	29 (2.4)	171 (0.9)	15	5.90	14
Belgium	15	24 (2)	403 (2.1)	11	16.79	25

^a^GDP: gross domestic product.

**Figure 4:**
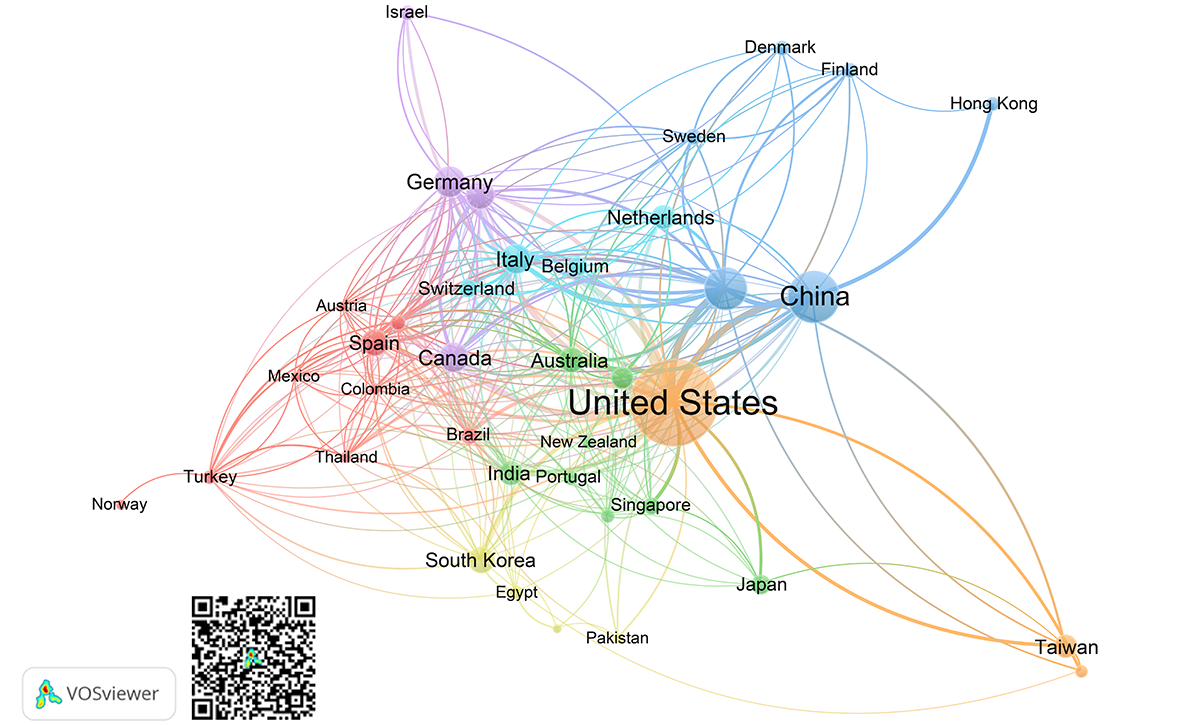
Bibliometric map created based on coauthorship analysis between countries with network visualization mode. The QR code can be used to open this figure in VOSviewer.

Figure 4 illustrates the distribution of the countries. This figure demonstrates how countries are establishing research networks and collaborating on the study of AI in intensive care. In VOSviewer, a country’s proximity to another indicates how strong their relatedness is. The stronger the link between 2 countries, the thicker the line. The results of coauthorship showed that the United States was the most affiliated country, being linked to 34 countries or territories with 247 times of coauthorship. The list was followed by the United Kingdom (28 links, 122 coauthorships), Italy (27 links, 93 coauthorships), China (26 links, 94 coauthorships), and others.

We listed the 15 most productive institutions based on the number of articles these institutions have published on AI in intensive care medicine (Table 4). Of the 15 institutions, 13 were in the United States, which further suggested the dominant role of the country in this research field. Of the 15 universities listed, 9 were in the top 100 best universities based on the World University Rankings 2022 [19].

**Table 4:** The top 15 most productive institutions based on the number of articles published on artificial intelligence in intensive care medicine.

Rank	Organization	TPi^a^	Citations, n	Citation per document	University ranking
1	Massachusetts Institute of Technology, Cambridge, United States	12	411	34.25	5
2	University of Michigan, Ann arbor, United States	11	77	7.00	24
3	Beth Israel Deaconess Medical Center, Boston, United States	9	596	66.22	—^b^
4	Harvard Medical School, Boston, United States	9	122	13.56	2
5	Mayo Clinic, Rochester, United States	9	41	4.56	—
6	University of Pennsylvania, Philadelphia, United States	8	130	16.25	13
7	Johns Hopkins University, Baltimore, United States	7	23	3.29	13
8	Georgia Institute of Technology, Atlanta, United States	7	347	49.57	45
9	Icahn School of Medicine at Mount Sinai, New York, United States	7	85	12.14	—
10	University of California San Francisco, San Francisco, United States	7	670	95.71	—
11	Emory University School of Medicine, Atlanta, United States	6	324	54.00	82
12	Peking University, Beijing, China	6	115	19.17	16
13	University of Pittsburgh, Pittsburgh, United States	6	139	23.17	—
14	Kuopio University Hospital, Kuopio, Finland	5	87	17.40	—
15	University of Chicago, Chicago, United States	5	139	27.80	10

^a^TPi: total publications of a given academic institution.

^b^Not available.

### Author Keywords

**Figure 5:**
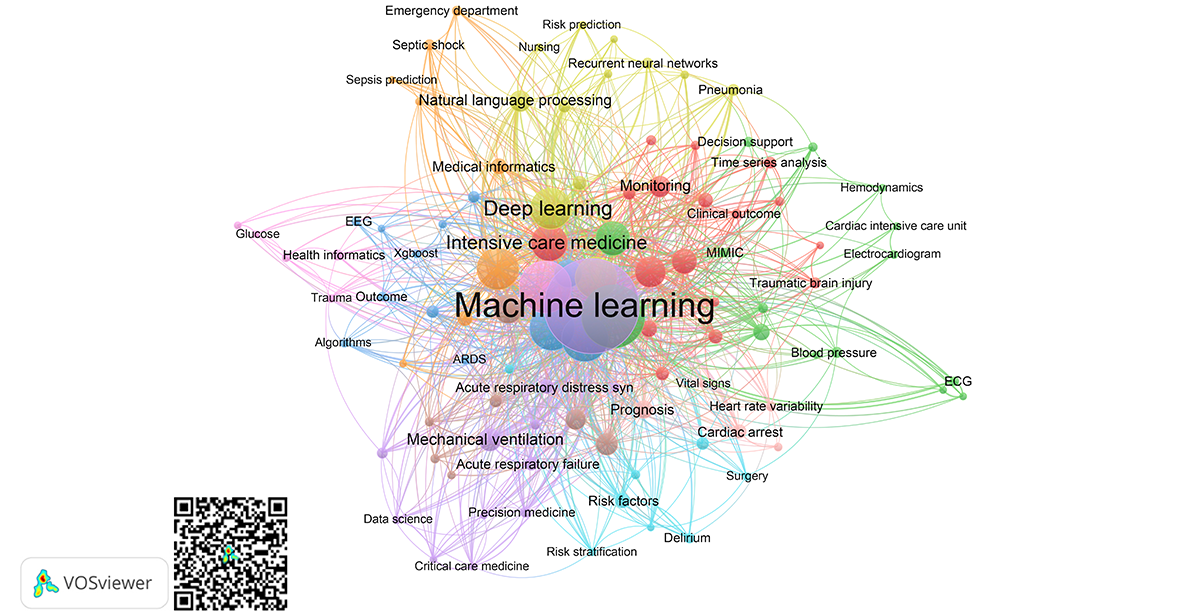
Bibliometric map created based on author keywords co-occurrence with network visualization mode. The QR code can be used to open the figure in VOSviewer. Colors show clustering. Keywords in the same cluster are of the same color. The circle size increases with the number of times a keyword is used. ARDS: acute respiratory distress syndrome; ECG: electrocardiogram; EEG: electroencephalogram.

**Figure 6:**
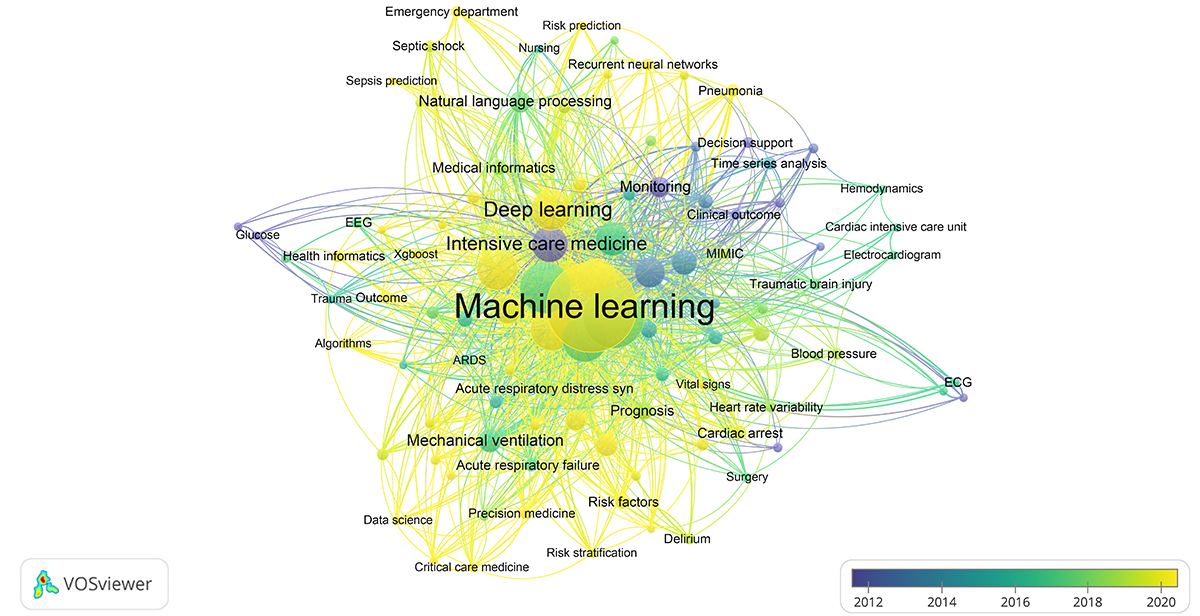
Bibliometric map created based on the co-occurrence of author keywords with overlay visualization mode. The color indicates the average publication year of the documents in which a keyword occurs. ARDS: acute respiratory distress syndrome; ECG: electrocardiogram; EEG: electroencephalogram.

Among the 2267 author keywords recorded, 1785 (78.73%) occurred only once, 230 (10.14%) occurred twice, and 252 (11.11%) occurred thrice. After relabeling synonymic words and congeneric phrases using the thesaurus file, 102 (4.49%) keywords met the threshold of a minimum of 5 occurrences for mapping in VOSviewer (Figure 5 and Figure 6; Table 5). *Machine learning* was the most frequently encountered keyword, with 347 occurrences and 809 links to other keywords. In the same cluster of *machine learning*, general terms included *big data* (9 occurrences, 15 links), *data science* (6 occurrences, 13 links), *prediction models* (5 occurrences, 10 links), and *clustering* (5 occurrences, 14 links). *Machine learning* co-occurred with ICU-related professional keywords, including *critical care medicine*, *acute respiratory distress syndrome*, *acute respiratory failure*, *endotracheal intubation*, *mechanical ventilation*, and *personalized medicine*.

**Table 5:** The 30 most frequently used keywords in articles on artificial intelligence in intensive care.

Keyword	Occurrences, n
Machine learning	347
Intensive care unit	172
Artificial intelligence	124
Critical care	102
Predictive analytics	97
Sepsis	86
COVID-19	85
Deep learning	84
Intensive care medicine	67
Decision support systems	60
Neural networks	50
Electronic health records	46
Mortality	42
Artificial neural networks	36
Mechanical ventilation	31
Predictive model	29
Natural language processing	27
Monitoring	26
Acute kidney injury	25
Prognosis	21
Data mining	17
Random forest	17
Medical informatics	16
Classification	15
Diagnosis	14
Risk factors	14
Acute respiratory distress syndrome	13
Cardiac arrest	13
Deterioration	13
Support vector machines	13

We categorized the keywords into 3 different categories based on the diseases in ICU, ML technologies used, and the function of ML in the research (Table 6). The top 5 diagnoses were sepsis, COVID-19, acute kidney injury, ARDS, and cardiac arrest. The top 5 AI technologies were ML, AI, deep learning, decision support systems, and neural networks. The top 5 functions were prediction (of clinical deterioration or mortality), mortality, monitoring, disease prognosis, and (disease phenotype) classification.

**Table 6:** Top author keywords in publications on artificial intelligence in critical care medicine.

Category			Frequency, n
**Disease**			
	Sepsis	86	
	COVID-19	85	
	Acute kidney injury	25	
	Acute respiratory distress syndrome	13	
	Cardiac arrest	13	
**Technology**			
	Machine learning	347	
	Artificial intelligence	124	
	Deep learning	84	
	Decision support systems	60	
	Neural networks	50	
**Function**			
	Prediction	97	
	Mortality	42	
	Monitoring	26	
	Prognosis	21	
	Classification	15	

### Citation Analysis

The 30 most cited articles among the 1198 articles on AI in intensive care medicine between 1986 and 2022 are presented in Table 7. The total number of citations and average number of citations per year are given. Of the 30 most cited articles, 10 (33%) were related to sepsis, one of the hottest topics in intensive care, and 11 (37%) articles were related to the use of AI technologies for the prediction of acute kidney injury, sepsis, hypotension diagnosis, clinical outcomes (mortality, survival, and ICU length of stay), and complications.

**Table 7:** The 30 most cited articles on artificial intelligence in intensive care unit ranked in the descending order of the number of citations.

Rank	Article	Citations, n	CPY^a^	Rank by CPY
1	Matthieu Komorowski, Leo A. Celi et al “The Artificial Intelligence Clinician Learns Optimal Treatment Strategies for Sepsis in Intensive Care” *Nature Medicine* (2018)	290	73	1
2	Shamim Nemati, Andre Holder et al “An Interpretable Machine Learning Model for Accurate Prediction of Sepsis in the ICU” *Critical Care Medicine*,46,4 (2018)	249	62	2
3	Tom J. Pollard, Alistair E. W. Johnson et al “The eICU Collaborative Research Database, a Freely Available Multi-Center Database for Critical Care Research” *Scientific Data*,5,1 (2018)	211	53	3
4	Thomas Desautels, Jacob Calvert et al “Prediction of Sepsis in the Intensive Care Unit With Minimal Electronic Health Record Data: a Machine Learning Approach” *JMIR Med Inform*,4,3 (2016)	203	34	7
5	Brendon P. Scicluna, Lonneke A. van Vught et al “Classification of Patients With Sepsis According to Blood Genomic Endotype: a Prospective Cohort Study” *The Lancet Respiratory Medicine*,5,10, (2017)	164	33	9
6	Alistair E. W. Johnson, Mohammad M. Ghassemi et al “Machine Learning and Decision Support in Critical Care” *Proceedings of the IEEE*,104,2, (2016)	162	27	11
7	Richard Dybowski, Peter Weller et al “Prediction of Outcome in Critically Ill Patients Using Artificial Neural Network Synthesised by Genetic Algorithm” *Lancet,*347,9009,4 (1996)	160	6	28
8	Romain Pirracchio, Maya L. Petersen et al “Mortality Prediction in Intensive Care Units With the Super ICU Learner Algorithm (SICULA): a Population-Based Study” *The Lancet Respiratory Medicine*,3,1 (2015)	160	23	15
9	Q. Li, G. D. Clifford “Dynamic Time Warping and Machine Learning for Signal Quality Assessment of Pulsatile Signals” *Physiological Measurement*,33,9 (2012a)	156	16	20
10	Anton Aboukhalil, Larry Nielsen et al “Reducing False Alarm Rates for Critical Arrhythmias Using the Arterial Blood Pressure Waveform” *Journal of Biomedical Informatics*,41,3,6 (2008)	151	11	23
11	Feras Hatib, Zhongping Jian et al “Machine-learning Algorithm to Predict Hypotension Based on High-Fidelity Arterial Pressure Waveform Analysis” *Anesthesiology*,129,4, 10 (2018)	147	37	5
12	U. Rajendra Acharya, Hamido Fujita et al “Automated Identification of Shockable and Non-Shockable Life-Threatening Ventricular Arrhythmias Using Convolutional Neural Network” *Future Generation Computer Systems*,79,2 (2018)	144	36	6
13	Qingqing Mao, Melissa Jay et al “Multicentre Validation of a Sepsis Prediction Algorithm Using Only Vital Sign Data in the Emergency Department, General Ward and ICU” *BMJ Open,*8,1 (2018)	134	34	8
14	Zhengping Che, Sanjay Purushotham et al “Interpretable Deep Models for ICU Outcome Prediction” *AMIA. Annual Symposium Proceedings* (2016)	132	22	17
15	Abbas K. Abbas, Konrad Heimann et al “Neonatal NonContact Respiratory Monitoring Based on Real-Time Infrared Thermography” *BioMedical Engineering Online,10,1,10* (2011)	126	11	21
16	Jacob S. Calvert, Daniel A. Price et al “A Computational Approach to Early Sepsis Detection“ *Computers in Biology and Medicine,74,*7 (2016)	116	19	18
17	Gilles Clermont, Derek C. Angus et al “Predicting Hospital Mortality for Patients in the Intensive Care Unit: a Comparison of Artificial Neural Networks with Logistic Regression Models” *Critical Care Medicine,*29,2 (2001)	112	10	24
18	David W. Shimabukuro, Christopher W. Barton et al “Effect of a Machine Learning-Based Severe Sepsis Prediction Algorithm on Patient Survival and Hospital Length of Stay: a Randomised Clinical Trial” BMJ *Open Respiratory Research,*4,1,11 (2017)	112	22	16
19	Jan Claassen, Kevin Doyle et al “Detection of Brain Activation in Unresponsive Patients with Acute Brain Injury” *New England Journal of Medicine,*380,26,6 (2019)	111	37	4
20	Sanjay Purushotham, Chuizheng Meng et al “Benchmarking Deep Learning Models on Large Healthcare Datasets” *Journal of Biomedical Informatics,*83,7 (2018)	105	26	12
21	Jay L. Kovner. Kyle A. Carey et al “The Development of a Machine Learning Inpatient Acute Kidney Injury Prediction Model” *Critical Care Medicine,*46,7 (2018)	105	26	13
22	Alexander Meyer, Dina Zverinski et al “Machine Learning for Real-Time Prediction of Complications in Critical Care: a Retrospective Study” *The Lancet Respiratory Medicine*,6,12 (2018)	101	25	14
23	Michel Dojat, Laurent Brochard et al “A Knowledge-Based System for Assisted Ventilation of Patients in Intensive Care Units” *International journal of clinical monitoring and computing 9,*4 (1992)	99	3	30
24	Nicos Maglaveras, Telemachos Stamkopoulos et al “An Adaptive Backpropagation Neural Network for Real-Time Ischemia Episodes Detection: Development and Performance Analysis Using the European ST-T Database” *IEEE Transactions on Biomedical Engineering,*45,7 (1998)	96	4	29
25	Allison Sutherland, Mervyn Thomas et al “Development and Validation of a Novel Molecular Biomarker Diagnostic Test for the Early Detection of Sepsis” *Critical Care,*15,3,6 (2011)	96	9	25
26	Hye Jin Kam, Ha Young Kim “Learning Representations for the Early Detection of Sepsis With Deep Neural Networks” *Computers in Biology and Medicine*,89,10 (2017)	96	19	19
27	Michelle M. Clark, Amber Hildreth et al “Diagnosis of Genetic Diseases in Seriously Ill Children by Rapid Whole-Genome Sequencing and Automated Phenotyping and Interpretation” *Science Translational Medicine,*11,489,4 (2019)	95	32	10
28	Subramani Mani, Asli Ozdas et al “Medical Decision Support Using Machine Learning for Early Detection of Late-Onset Neonatal Sepsis” *Journal of the American Medical Informatics Association*,21,2,3 (2014)	89	11	22
29	K. Ashwin Kumar, Yashwardhan Singh et al “Hybrid Approach Using Case-Based Reasoning and Rule-Based Reasoning for Domain Independent Clinical Decision Support in ICU” *Expert Systems with Applications*,36,1 (2009)	86	7	27
30	Qiao Li, Gari D. Clifford “Signal Quality and Data Fusion For False Alarm Reduction in the Intensive Care Unit” *Journal of Electrocardiology*,45,6,11 (2012b)	85	9	26

^a^CPY: citations per year.

## Discussion

Our study used a bibliometric method to analyze AI in intensive care medicine research by examining publication output, the growth of research interest, preferred journals, leading countries, international collaboration, top institutions, author keywords, and citation analysis.

### Publication Output and Growth of Research Interest

Since the oldest publication in 1986, publications on AI in intensive care medicine had a slow growth for 30 years. The turning point appeared in 2018 when there was a significant growth in the interest in AI in intensive care medicine. This lags 6 years behind the rapid growth in the interest in AI in general medicine that started in 2012 [20]. This is likely owing to concerns regarding the safety and accountability of the AI model in critically ill patients. The AI technologies that emerged from 2014 to 2018 such as autonomous robots, voice recognition, neural networks, and ML provided unprecedented opportunities to predict, diagnose, and manage diseases. Large public critical care databases such as MIMIC and eICU became readily available to researchers in 2016 and 2018 [5,21]. Advancements in AI technology and large databases have contributed to the steep increase in annual publications since 2018. Based on the publication trend, it is anticipated that the annual publications will continue to increase.

### Preferred Journals

Of the top 15 productive journals, 9 (60%) had a CiteScore of ≥5, which suggested that critical care medicine–related AI research is favored by the top journals in critical care and medical informatics. These include *Critical Care Medicine*, *Critical Care and IEEE Journal of Biomedical and Health Informatics*. Authors who want to publish critical care medicine–related AI research could first consider the top productive journals listed. CiteScore, an Elsevier-Scopus alternative to the Clarivate Analytics Impact Factor, is a metric for assessing journal impact based on citation data from the Scopus database. However, CiteScore is not the only factor considered when deciding which journal to publish in. Authors should consider the ability of the journal to disseminate the research work to the right audience and contribute to the progression of the field [8].

### Leading Countries, International Collaboration, and Top Institutions

The fact that 9 of the top 15 productive institutions are among the top 100 best universities demonstrated that AI in critical care medicine has received attention at the top universities worldwide. Authors could consider joint research with those institutions or apply for their visiting scholar or educational programs.

When the distribution of publications by countries was examined, high-income countries were the leading force in critical care medicine–related AI research. The top 10 most productive countries are among the top 25 in terms of world gross domestic product, which suggested that the economic power of the countries affects the productivity of their publications. This result is the same as that of the bibliometric research on many other medical subjects [13,22,23]. About 60% of the global publications were contributed by the United States, China, and the United Kingdom, indicating that these 3 countries contributed the most to AI in critical care research. These countries also had the highest citations, although China had relatively low citations per document compared with the United States and the United Kingdom.

The number of citations was lower than that of other research hot spots in critical care medicine [24]. This is likely because of the limitations of AI-related studies in critical care, which include low maturity of AI in real-world application [25] and a lack of external validation process, prospective evaluation, and clear protocols to examine the reproducibility of AI solutions [26].

Coauthorship analysis revealed that the United States, the United Kingdom, Italy, and China were the most affiliated countries with >90 coauthorships. The diversity of research partners, a high proportion of foreign postgraduates or visiting scholars, and adequate research funding were all factors that contributed to improved international collaboration. To ensure the sustainability of international collaboration, a flexible and stable research policy is also crucial [8].

### Author Keywords and Citation Analysis

According to the author keywords in the identified categories, the top domains of disease covered in critical care medicine–related AI research were sepsis, COVID-19, acute kidney injury, ARDS, and cardiac arrest. These most prevalent ICU conditions have become a popular target for AI algorithms.

The top functions of AI include the prediction of clinical deterioration or disease evolution or mortality, monitoring, disease prognosis, and disease phenotype or subtype classification. The literature reported other important functions such as disease identification and guiding decision-making (reinforcement learning) [26]. The keywords of high occurrence in the titles of the 30 most cited articles include *sepsis*, *prediction*, *early detection*, and *clinical decision support*. Keyword analysis showed that *machine learning* was frequently related to respiratory diseases, especially COVID-19. The most widely used AI technologies in critical care include ML, deep learning, decision support systems, and neural networks.

### Limitations

Our research provided a general review of the research trends and hot spots in critical care medicine–related AI research, highlighted the most productive countries and academic institutions to facilitate potential collaboration, and provided directions for future research. However, this bibliometric study has several limitations.

First, even though we included the most widely used AI technologies and made an effort to be specific about AI-related terms in the search keywords (eg, *neural network*, *machine learning*, *deep learning*, and *natural language processing*), they were still quite general and did not include all AI technologies. Because of restricting the search to only those keywords in the title, abstract, and keywords, the search result may not have covered all AI in critical care medicine–related studies available on Scopus. Furthermore, because of missing author keyword information, the co-occurrence analysis of author keywords included only 74.4% (891) of the 1198 articles.

Second, our study used only the Scopus database, which is the largest abstract and citation database that we think should be sufficient for our analysis. Bibliometric analysis using multiple data sources such as the Web of Sciences, PubMed, and Google Scholar will be more comprehensive. For instance, Web of Science has a feature called “hot paper” that is not available in Scopus, which automatically displays the most popular articles in the field [27]. The hot paper feature displays important papers that were identified immediately after publication, as indicated by a sharp rise in the number of citations [8].

Finally, we did not include papers published in the form of conference papers, reviews, editorials, notes, letters, book chapters, short surveys, or data papers owing to concerns about the low clinical readiness of those publications. As a result, we may have missed relevant studies published in forms other than articles. Because AI technology is a cutting-edge and rapidly evolving research area, papers published in conference proceedings and letters may have reviewed the latest updates in the field.

Future bibliometric analyses could use more specific AI technology terms in the research keywords; use other databases such as Web of Sciences, PubMed, and Google Scholar; and include conference papers in the type of articles to explore more potential papers.

### Conclusions

Our study has provided an overview of the research trends of AI in critical care medicine based on 1198 publications retrieved from the Scopus database. Publication growth was rapid in the last 5 years and is expected to further increase. We have reviewed countries and academic institutions (eg, the United States, China, and the United Kingdom) that have a substantial number of publications and solid international collaborations. This provides potential collaboration opportunities to other countries, especially to low- and middle-income countries that lack AI technologies but have an increasing demand for health care resources.

We have discussed several conditions in critical care that are currently actively explored using AI technology, such as sepsis, COVID-19, acute kidney injury, ARDS, and cardiac arrest. AI research hot spots in critically ill patients involve detecting clinical deterioration, monitoring, predicting disease evolution, mortality, disease prognosis, and classifying disease phenotypes or subtypes. The most widely used AI technologies in critical care research are ML, deep learning, decision support systems, and neural networks. The 30 articles that received the most citations between 1986 and 2022 have been listed in detail.

AI research on critical care is a rising hot spot in both critical care and AI research, with potential applications being demonstrated across various domains of critical care medicine. However, the development and implementation of AI solutions still face many challenges. AI research application in clinical settings has been constrained by a lack of external validation processes, prospective evaluation, and clear protocols to examine the reproducibility of AI solutions. For AI-based clinical research to be sufficiently convincing for routine critical care practice, collaborative research efforts are needed to increase the maturity and robustness of AI-driven models [26].

## Abbreviations

AI: artificial intelligence
ARDS: acute respiratory distress syndrome
ICU: intensive care unit
ML: machine learning
